# Association of vitamin D status with coronary artery disease in postmenopausal women

**DOI:** 10.1097/MD.0000000000019544

**Published:** 2020-03-13

**Authors:** Rui Xu, Yan-Yan Li, Ling-Ling Ma, Hong-Ni Yang

**Affiliations:** a Gerontology center; b Department of cardiac surgery, People's Hospital of Xinjiang Uygur Autonomous Region, Urumqi, China.

**Keywords:** 25-hydroxyvitamin D, coronary artery disease, postmenopausal women, vitamin D deficiency

## Abstract

The relationship between coronary artery disease (CAD) and low serum 25-hydroxyvitamin D (25(OH)D) levels has emerged. Postmenopausal (PM) women are at increased risk of CAD and vitamin D (VitD) deficiency.

To investigate the relationship between CAD and VitD levels in PM women.

This case–control study included 93 consecutive female patients aged 50 to 79 years old undergoing coronary angiography for evaluation of CAD and 119 age-matched controls. Serum 25(OH)D concentrations were classed as adequate (serum 25(OH)D: ≥20 ng/mL); insufficient (serum 25(OH)D: 10 to <20 ng/mL); and deficient (serum 25(OH)D: <10 ng/mL). Major cardiovascular risk factors were also explored.

CAD occurred in 67/127 (52.8%) patients with VitD deficiency; 21/66 (31.8%) patients that were VitD insufficient; and in 5/19 (26.3%) patients with adequate VitD levels. Multivariate regression analysis suggested that a deficiency of VitD increased CAD (odds ratio = 2.891; 95% confidence interval = 1.459–7.139, *P* < .001).

VitD deficiency should be evaluated in PM women as a possible cause of CAD.

## 1 Introduction

Vitamin D (VitD) is a fat-soluble prohormone required for Ca^2+^ metabolism, bone growth, and nonskeletal processes.^[1]^ Emerging evidence supports a link between VitD deficiency and cardiovascular disease including heart failure, peripheral vascular disease, dyslipidemia, diabetes, hypertension, and coronary artery disease (CAD).^[2–4]^

CAD is a major cause of morbidity and mortality. VitD receptors are present in the cardiovascular system and epidemiological studies highlight the association of VitD status to CAD risk.^[5]^ In a meta-analysis (MA) of 19 prospective studies that included 65,994 participants, an inverse association between circulating VitD and cardiovascular disease was reported.^[6]^ In direct contrast to these studies, the assessment of 813 men showed no VitD association with cardiovascular disease.^[7]^ A MA assessing the effects of VitD supplements to cardiovascular outcomes also showed minimal association.^[8]^

VitD deficiency is common during menopause, higher latitudes, reduced sunlight exposure, skin pigmentation, and a low-VitD diet.^[9,10]^ The risk of VitD deficiency in older women is higher than aged men, thought to be due to reduced estrogen production.^[11]^ Here, we investigated the association of VitD with CAD in postmenopausal (PM) women.

## 2 Materials and methods

This study was approved by the Ethics Committee of People's Hospital of Xinjiang Uygur Autonomous Region. It was conducted according to the standards of the Declaration of Helsinki. Written informed consent was obtained from all of the participants.

### 2.1 Study population

The patient population included 93 CAD patients and 119 age-matched female controls admitted to the Department of Geriatrics, People's Hospital of Xinjiang Uygur Autonomous Region, China, from January 1, 2018 to August 31, 2018. This unit primarily admits elderly patients with respiratory and cardiovascular disease. The inclusion criteria were: PM women; aged ≥50 years; over 50% angiographic stenosis of at least 1 coronary artery; not receiving Ca^2+^ or VitD. Exclusion criteria: metabolic VitD disorders, thyrotoxicosis, hyperparathyroidism, renal failure, or malignancies liver associated disease.

### 2.2 Cardiovascular risk factors

Height, weight, and body mass index (BMI) were calculated (kg/m^2^). Hypertension was defined as systolic blood pressure ≥140 mm Hg or an average diastolic blood pressure ≥90 mm Hg recorded in a minimum of 2 independent medical examinations. Diabetes was defined as fasting plasma glucose levels ≥7.0 mmol/L (126 mg/dL) or normal glucose values ≥11.1 mmol/L (200 mg/dL). Those who smoked at least once per day were classified as “smokers," and those who consumed at least 1 drink per week were classified as “drinkers."

### 2.3 Blood assessments

Biochemical parameters were measured from the peripheral blood of fasted patients. Total cholesterol, low density lipoprotein-cholesterol, high density lipoprotein-cholesterol, and triglycerides were measured using commercial kits. Serum 25-hydroxyvitamin D (25(OH)D) was measured via chemiluminescence microparticle immunoassays using an Architect system. The intra/interassay coefficients of variation were 4.1% and 5.7%, respectively. VitD deficiency was classed as 25(OH)D < 10 ng/mL; VitD insufficiency was classed as 10 to <20 ng/mL. Normal VitD levels are 25(OH)D ≥20 ng/mL.

### 2.4 Statistics

Data were compared using SPSS (SPSS, Inc., Chicago, IL). version 20.0. Gaussian distributions were used to measure continuous variables which are presented as the mean ± standard deviation. Non-Gaussian distributions are shown as median values in the 25th and 75th percentiles. Normal distributions were verified using the Kolmogorov–Smirnov test. Continuous variables was calculated by the unpaired t test. Categorical variables was calculated Chi-squared test. Intergroup differences were compared using unpaired *t* test. Chi-squared tests were used for categorical variables and logistic regression analysis was used to identify independent CAD risk factors. *P* < .05 indicated statistical significance.

## 3 Results

Table 1 shows the patient characteristics of the study population. The cohort included 212 PM females (mean age 67.32 ± 6.51 years). Based on VitD status, subjects were classed as adequate (n = 19), insufficient (n = 66), and deficient (n = 127). The mean age of the groups significantly differed but all other demographics and laboratory measurements were comparable (Table 2).

**Table 1:**
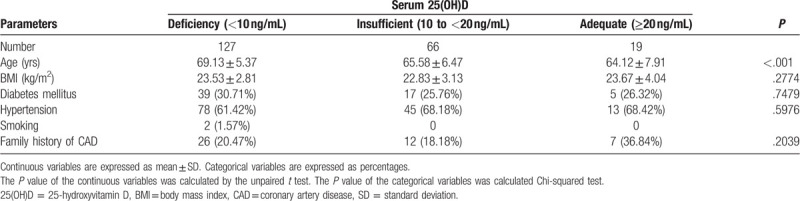
Demographics of the study population, mean ± SD, n (%).

**Table 2:**
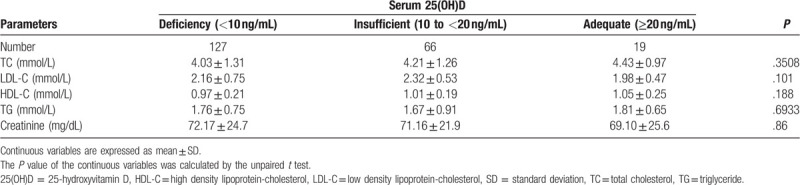
Laboratory parameters of the patients, mean ± SD.

**Figure 1:**
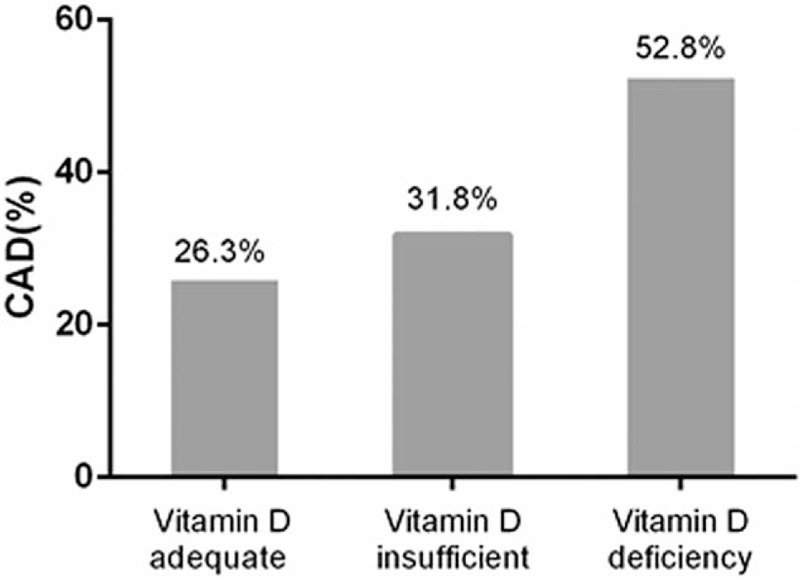
Prevalence of CAD increases gradually in patients with vitamin D adequate, vitamin D insufficient, and vitamin D deficiency with a significant level (*P* = .0057). CAD = coronary artery disease.

Prevalence of CAD in PM women based on VitD status is shown in Figure 1. CAD occurred in 5/19 (26.3%) patients in the VitD adequate group; 21/66 (31.8%) patients in the VitD insufficient group; and 67/127 (52.8%) patients in the VitD deficient group. CAD prevalence increased from adequate to deficient groups (*P* = .006) (Fig. 1).

Multivariate logistic regression analysis was used to investigate the association between VitD status and CAD after adjusting for age (base model), and risk factors (full model) (Table 3). Multivariate regression analysis of the full model revealed that VitD deficiency increased CAD prevalence as an independent correlate (odds ratio = 2.891; 95% confidence interval = 1.459–7.139, *P* < .001) in addition to age, BMI, diabetes, and hypertension, smoking, family history of CAD, TC, LDL-C, HDL-C, TG, and creatinine.

**Table 3:**
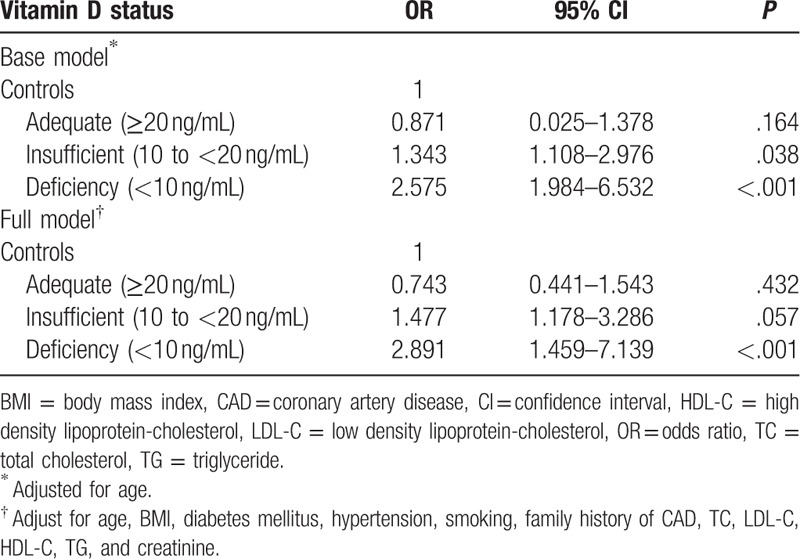
Multivariate regression analysis between vitamin D status and coronary artery disease.

## 4 Discussion

We report that PM women with VitD deficiency/insufficiency have a higher prevalence of CAD compared to patients with normal VitD levels. From logistic regression analysis, 25(OH)D was an independent risk factor for CAD. The risk of CAD was 3-fold higher in those with 25(OH)D < 10 ng/mL compared to those with 25(OH)D ≥ 20 ng/mL. Serum 25 (OH)D levels are thus clinically relevant to CAD prevalence.

VitD deficient patients had a CAD prevalence of 52.8%, and those with VitD insufficiency had a prevalence of 31.8%. These results are similar to prior study from India that reported values of 51.2% and 44.6%, respectively.^[12]^ Study from Mexico reported a CAD prevalence of 57.6% and 21.2%, respectively.^[13]^ Moreover, the prevalence of hypovitaminosis D is more frequent in PM women compared to age-matched men.^[14]^ These findings indicate that hypovitaminosis D is a common occurrence in PM women with CAD. Awareness and treatment for VitD deficiency are thus crucial interventions to improve CAD prognosis.

In a study performed in Saudi Arabia, the relationship between CAD and VitD Status was examined in 130 CAD cases and 195 age–sex matched controls. A direct association between VitD Status and CAD was shown.^[15]^ Studies performed in individuals with no history of circulatory diseases demonstrated that the risk of cardiovascular incidents were 1.5-fold higher in patients with 25(OH)D ≤ 15 ng/mL.^[16]^ In contrast, Messenger et al^[7]^ found no association of VitD status with CAD in 813 men with VitD deficiency (>15 ng/mL) compared to those with normal VitD levels (<30 ng/mL). However, VitD deficiency has various classifications making interstudy comparisons challenging. We present data from a Chinese cohort but further studies in other ethnic groups are required to further our findings.

While VitD deficiency and CAD have been described extensively in the literature, their precise relationship remains unclear. Atherosclerosis in coronary arteries is critical to the pathogenesis of CAD.^[17]^ Inflammation plays a key role in atherosclerosis and VitD deficiency is a known cause of inflammation.^[18,19]^ Low VitD levels directly increase C-reactive protein (CRP) synthesis and the protective effects of VitD to CRP are evidenced by the distribution of VitD receptors in the vascular walls.^[20]^ In vivo studies have shown that a loss of VitD receptor expression influences cardiac function. VitD knockout mice tend to develop left ventricular hypertrophy and heart failure.^[21]^ VitD deficiency negatively affects the cardiovascular system through activation of the renin–angiotensin–aldosterone system.^[22]^ Further studies should now investigate the common pathophysiological links between VitD deficiency and CAD.

This study had some limitations. First, our analysis was observational and no follow-up studies were performed. Secondly, the sample size was small and required verification in larger study cohorts. Information on VitD receptor expression, inflammatory cytokines, and sunlight exposure should also be included to identify the mechanisms associated with VitD deficiency and subsequent CAD.

In this study, we investigated the association between VitD deficiency and CAD in PM women and revealed an association. Our data also suggest that VitD deficiency is an appropriate diagnostic tool for CAD assessments. This would promote the earlier identification and treatment of CAD, which is an important part of CAD management in PM women.

## Author contributions

Conception and design of the research: Rui Xu, Hong-Ni Yang; Acquisition of data and Analysis and interpretation of the data: Yan-Yan Li, Ling-Ling Ma; Statistical analysis: Rui Xu; Writing, review and editing of the manuscript: Rui Xu.
